# 3-Mesityl-2-oxo-1-oxaspiro­[4.4]non-3-en-4-yl 4-chloro­benzoate

**DOI:** 10.1107/S1600536809006503

**Published:** 2009-03-06

**Authors:** Ming-Hua Ji, Min Xia, Guo-Nian Zhu, Jin-Hao Zhao

**Affiliations:** a Institute of Science, Zhejiang Sci_tec University, Hangzhou 310032, People’s Republic of China; b College of Agriculture and Biotechnology, Zhejiang University, Hangzhou 310029, People’s Republic of China

## Abstract

The title compound, C_24_H_23_ClO_4_, is a potent insecticide and miticide. The five-membered cyclo­pentane ring displays an envelope conformation with the atom at the flap position 0.611 (2) Å out of the mean plane formed by the other four atoms. The furan ring makes dihedral angles of 71.3 (2) and 81.9 (2)°, respectively, with the 2,4,6-trimethyl­phenyl and 4-chloro­phenyl rings. The dihedral angle between the two benzene rings is 76.6 (1)°. In the crystal, mol­ecules are linked through weak inter­molecular C—H⋯O hydrogen bonds, forming chains running along the *c* axis.

## Related literature

For a related insecticide, see: Bayer, (1995 ▶). For a related methyl­butyrate structure, see: Yu *et al.* (2009 ▶). For the extinction correction, see: Larson (1970 ▶).
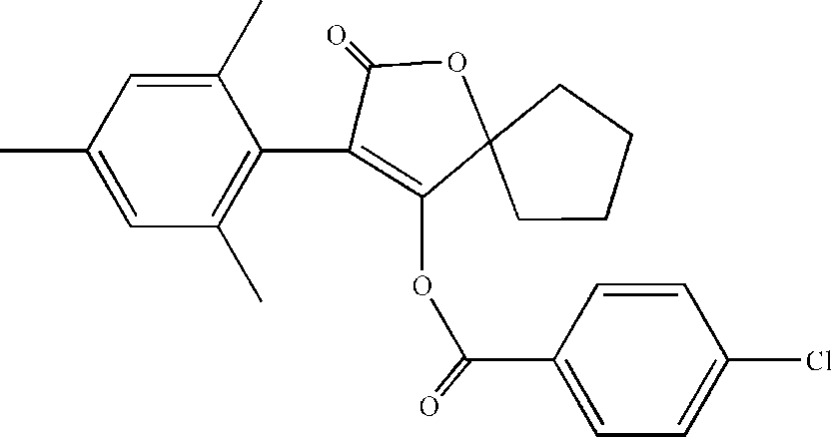


## Experimental

### 

#### Crystal data

C_24_H_23_ClO_4_
*M* *_r_* = 410.90
Monoclinic, 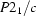

*a* = 6.4880 (2) Å
*b* = 22.9397 (8) Å
*c* = 14.6305 (6) Å
β = 91.533 (1)°
*V* = 2176.72 (14) Å^3^
*Z* = 4
Mo *K*α radiation
μ = 0.20 mm^−1^
*T* = 296 K
0.62 × 0.48 × 0.34 mm

#### Data collection

Rigaku R-AXIS RAPID diffractometer
Absorption correction: multi-scan (*ABSCOR*; Higashi, 1995 ▶) *T*_min_ = 0.867, *T*_max_ = 0.934
33470 measured reflections
4926 independent reflections
3235 reflections with *F* ^2^ > 2σ(*F* ^2^)
*R*_int_ = 0.030

#### Refinement

*R*[*F* ^2^ > 2σ(*F* ^2^)] = 0.037
*wR*(*F* ^2^) = 0.092
*S* = 1.00
4926 reflections
263 parameters
H-atom parameters constrained
Δρ_max_ = 0.14 e Å^−3^
Δρ_min_ = −0.17 e Å^−3^

### 

Data collection: *PROCESS-AUTO* (Rigaku, 1998 ▶); cell refinement: *PROCESS-AUTO*; data reduction: *CrystalStructure* (Rigaku/MSC, 2004 ▶); program(s) used to solve structure: *SIR97* (Altomare *et al.*, 1993 ▶); program(s) used to refine structure: *CRYSTALS* (Betteridge *et al.*, 2003 ▶); molecular graphics: *ORTEP-3 for Windows* (Farrugia, 1997 ▶); software used to prepare material for publication: *CrystalStructure*.

## Supplementary Material

Crystal structure: contains datablocks global, I. DOI: 10.1107/S1600536809006503/si2154sup1.cif

Structure factors: contains datablocks I. DOI: 10.1107/S1600536809006503/si2154Isup2.hkl

Additional supplementary materials:  crystallographic information; 3D view; checkCIF report

## Figures and Tables

**Table 1:** Hydrogen-bond geometry (Å, °)

*D*—H⋯*A*	*D*—H	H⋯*A*	*D*⋯*A*	*D*—H⋯*A*
C24—H242⋯O2^i^	0.97	2.57	3.475 (2)	155

Symmetry code: (i) 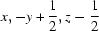.

**Fig. 1.**
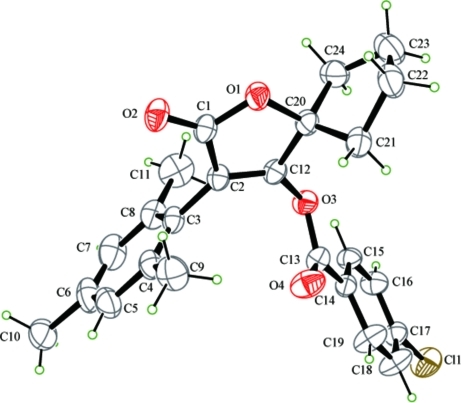
The molecular structure of (I), showing the atom-labelling scheme. Displacement ellipsoids are drawn at the 30% probability level.

**Fig. 2.**
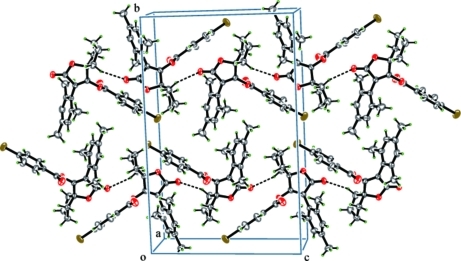
A packing diagram of the title compound, viewed along the a axis.

## supplementary crystallographic information

### Comment

4-hydroxyl-3-(2,4,6-trimethylphenyl)-1-oxaspiro[4,4]non-3-en-2-one (HTPO) is a key intermediate of Spiromesifen, which is an efficient insecticide and miticide, developed by Bayer company (Bayer, 1995). As part of our continuing interest in the design and synthesis of the new insecticide and miticide, we have isolated the title compound (Fig. 1), by the condensation reaction of 4-chlorophenyl- acetylchloride and HTPO as colorless crystals. The molecule contains two six-membered rings and two five-membered rings. Atoms C1, C2, C12, C20, O1 and O2 are coplanar, the largest deviation being 0.011 (11) Å for O1. As expected, C2=C12, C1=O2 and C13=O4 are typical double bonds with bond distances of 1.317 (2), 1.202 (2) and 1.195 (3) Å suggests that C2, C12 and C13 atoms are sp^2^ hybridized. The bond distance of C1—C2 is 1.475 (2) Å, suggesting that the carbonyl group on C1 has formed a conjugate system with double bond on C2 and C12. In the crystal, molecules are linked through weak intermolecular C—H···O hydrogen bonds (Table 1), forming chains running along the *c* axis (Fig. 2), in contrast to the related 3-mesityl-2-oxo-1-oxaspiro[4.4]non-3-en-4-yl 2-(chlorophenyl)-3-methyl- butyrate structure, where intermolecular C—H···Cl hydrogen bonds forming chains along the screw axis direction *b* (Yu *et al.* 2009).

### Experimental

4-hydroxyl-3-(2,4,6-trimethylphenyl)-1-oxaspiro[4,4]non-3-en-2-one (0.272 g, 1 mmol) and triethylamine (0.152 g, 1.5 mmol) were dissolved in dry dichloromethane (15 ml) with stirring. 4-chlorophenylacetyl chloride (0.210 g, 1.2 mmol) was added dropwise to the mixture in a water bath. The mixture was stirred at 293–298 K for 5 h, and then 1% aqueous HCl was added. The organic layer was washed to neutral with water and dried *via* Na_2_SO_4_. After filtered and concentrated, the organic residue was purified by silica gel column chromatography, eluted with ethyl acetate-petroleum ether (1:3. *v*/*v*) to give a white solid (yield 81%, 0.334 g), which was then recrystallized from acetone/ethanol (1:2, *v*/*v*) to give colourless blocks.

### Refinement

The H atoms were geometrically placed (C—H = 0.93–0.98 Å) and refined as riding with *U*_iso_(H) = 1.2*U*_eq_(C) or 1.5*U*_eq_(methyl C). The methyl group was allowed to rotate, but not to tip, to best fit the electron density.

### Crystal data

**Table:**

C_24_H_23_ClO_4_	*F*(000) = 864.00
*M**_r_* = 410.90	*D*_x_ = 1.254 Mg m^−^^3^
Monoclinic, *P*2_1_/*c*	Mo *K*α radiation, λ = 0.71075 Å
Hall symbol: -P 2ybc	Cell parameters from 20338 reflections
*a* = 6.4880 (2) Å	θ = 3.0–27.4°
*b* = 22.9397 (8) Å	µ = 0.20 mm^−^^1^
*c* = 14.6305 (6) Å	*T* = 296 K
β = 91.533 (1)°	Block, colorless
*V* = 2176.72 (14) Å^3^	0.62 × 0.48 × 0.34 mm
*Z* = 4	

### Data collection

**Table:**

Rigaku R-AXIS RAPID diffractometer	3235 reflections with *F*^2^ > 2σ(*F*^2^)
Detector resolution: 10.00 pixels mm^-1^	*R*_int_ = 0.030
ω scans	θ_max_ = 27.5°
Absorption correction: multi-scan (*ABSCOR*; Higashi, 1995)	*h* = −8→7
*T*_min_ = 0.867, *T*_max_ = 0.934	*k* = −29→29
33470 measured reflections	*l* = −18→18
4926 independent reflections	

### Refinement

**Table:**

Refinement on *F*^2^	*w* = 1/[0.0002*F*_o_^2^ + 1.45σ(*F*_o_^2^)]/(4*F*_o_^2^)
*R*[*F*^2^ > 2σ(*F*^2^)] = 0.037	(Δ/σ)_max_ < 0.001
*wR*(*F*^2^) = 0.092	Δρ_max_ = 0.14 e Å^−^^3^
*S* = 1.00	Δρ_min_ = −0.17 e Å^−^^3^
4926 reflections	Extinction correction: Larson (1970), equation 22
263 parameters	Extinction coefficient: 695 (30)
H-atom parameters constrained	

### Special details

**Table:**

Refinement. Refinement using all reflections. The weighted *R*-factor (*wR*) and goodness of fit (*S*) are based on *F*^2^. *R*-factor (gt) are based on *F*. The threshold expression of *F*^2^ > 2.0 σ(*F*^2^) is used only for calculating *R*-factor (gt).

### Fractional atomic coordinates and isotropic or equivalent isotropic displacement parameters (Å^2^)

**Table:**

	*x*	*y*	*z*	*U*_iso_*/*U*_eq_	
Cl1	0.25024 (8)	0.45321 (2)	−0.01486 (4)	0.0960 (2)	
O1	0.83337 (16)	0.19253 (4)	0.49303 (8)	0.0589 (3)	
O2	0.83728 (17)	0.23623 (5)	0.62950 (8)	0.0671 (3)	
O3	0.64511 (14)	0.29954 (4)	0.33174 (6)	0.0563 (3)	
O4	0.31480 (18)	0.29640 (6)	0.37120 (10)	0.0822 (5)	
C1	0.7983 (2)	0.23874 (6)	0.54880 (12)	0.0531 (5)	
C2	0.7122 (2)	0.28760 (6)	0.49455 (12)	0.0493 (4)	
C3	0.6530 (2)	0.34424 (6)	0.53503 (11)	0.0517 (4)	
C4	0.4814 (2)	0.34712 (6)	0.59006 (12)	0.0617 (5)	
C5	0.4308 (3)	0.40037 (8)	0.62872 (12)	0.0815 (7)	
C6	0.5482 (4)	0.45001 (8)	0.61565 (16)	0.0906 (8)	
C7	0.7148 (3)	0.44552 (8)	0.56064 (17)	0.0903 (7)	
C8	0.7716 (2)	0.39386 (6)	0.51955 (12)	0.0664 (5)	
C9	0.3514 (2)	0.29404 (8)	0.60759 (14)	0.0839 (7)	
C10	0.4933 (4)	0.50730 (9)	0.66180 (18)	0.1028 (11)	
C11	0.9564 (2)	0.39222 (8)	0.45970 (17)	0.0990 (8)	
C12	0.7031 (2)	0.26889 (6)	0.40947 (12)	0.0498 (4)	
C13	0.4412 (2)	0.31350 (6)	0.31964 (12)	0.0546 (5)	
C14	0.4018 (2)	0.34881 (6)	0.23706 (11)	0.0479 (4)	
C15	0.5560 (2)	0.37213 (6)	0.18689 (12)	0.0557 (5)	
C16	0.5109 (2)	0.40460 (6)	0.10976 (12)	0.0653 (5)	
C17	0.3095 (2)	0.41340 (6)	0.08306 (12)	0.0599 (5)	
C18	0.1545 (2)	0.38918 (8)	0.13064 (14)	0.0864 (7)	
C19	0.1998 (2)	0.35689 (8)	0.20804 (14)	0.0813 (6)	
C20	0.7718 (2)	0.20699 (6)	0.39878 (11)	0.0526 (4)	
C21	0.6061 (2)	0.16400 (6)	0.36752 (12)	0.0650 (5)	
C22	0.7311 (3)	0.10964 (6)	0.34622 (13)	0.0819 (6)	
C23	0.9180 (3)	0.13309 (9)	0.29939 (14)	0.0926 (7)	
C24	0.9516 (2)	0.19490 (6)	0.33625 (12)	0.0718 (6)	
H5	0.3147	0.4028	0.6644	0.098*	
H7	0.7933	0.4787	0.5504	0.108*	
H15	0.6928	0.3660	0.2050	0.067*	
H16	0.6166	0.4205	0.0760	0.078*	
H18	0.0182	0.3944	0.1111	0.104*	
H19	0.0936	0.3405	0.2409	0.098*	
H91	0.2999	0.2786	0.5504	0.101*	
H92	0.2377	0.3047	0.6448	0.101*	
H93	0.4337	0.2650	0.6387	0.101*	
H101	0.3526	0.5060	0.6804	0.123*	
H102	0.5823	0.5131	0.7145	0.123*	
H103	0.5104	0.5389	0.6196	0.123*	
H111	1.0216	0.3547	0.4648	0.119*	
H112	0.9133	0.3989	0.3973	0.119*	
H113	1.0523	0.4220	0.4788	0.119*	
H211	0.5096	0.1565	0.4156	0.078*	
H212	0.5318	0.1781	0.3135	0.078*	
H221	0.7709	0.0891	0.4019	0.098*	
H222	0.6533	0.0836	0.3060	0.098*	
H231	1.0375	0.1090	0.3136	0.111*	
H232	0.8938	0.1340	0.2337	0.111*	
H241	1.0814	0.1973	0.3705	0.086*	
H242	0.9517	0.2227	0.2863	0.086*	

### Atomic displacement parameters (Å^2^)

**Table:**

	*U*^11^	*U*^22^	*U*^33^	*U*^12^	*U*^13^	*U*^23^
Cl1	0.1083 (4)	0.0976 (3)	0.0808 (4)	0.0141 (2)	−0.0245 (3)	0.0319 (3)
O1	0.0795 (7)	0.0537 (6)	0.0429 (7)	0.0070 (5)	−0.0072 (5)	0.0039 (5)
O2	0.0907 (8)	0.0672 (7)	0.0425 (8)	−0.0056 (5)	−0.0117 (6)	0.0056 (6)
O3	0.0610 (6)	0.0657 (6)	0.0422 (7)	0.0093 (5)	0.0000 (5)	0.0114 (5)
O4	0.0608 (7)	0.1034 (11)	0.0823 (11)	−0.0012 (7)	0.0019 (7)	0.0253 (9)
C1	0.0638 (10)	0.0529 (9)	0.0423 (11)	−0.0086 (7)	−0.0043 (8)	0.0043 (8)
C2	0.0584 (9)	0.0474 (8)	0.0419 (10)	−0.0054 (6)	−0.0011 (7)	0.0040 (7)
C3	0.0668 (10)	0.0462 (8)	0.0417 (10)	−0.0040 (7)	−0.0053 (8)	0.0022 (7)
C4	0.0803 (11)	0.0605 (10)	0.0442 (11)	0.0015 (8)	−0.0008 (9)	−0.0006 (8)
C5	0.1075 (15)	0.0836 (13)	0.0534 (13)	0.0250 (11)	0.0025 (11)	−0.0061 (11)
C6	0.152 (2)	0.0569 (12)	0.0618 (15)	0.0231 (13)	−0.0212 (14)	−0.0090 (10)
C7	0.1344 (19)	0.0487 (10)	0.0867 (17)	−0.0122 (11)	−0.0215 (14)	0.0013 (11)
C8	0.0826 (12)	0.0518 (10)	0.0642 (13)	−0.0084 (8)	−0.0087 (10)	0.0072 (9)
C9	0.0858 (13)	0.0926 (13)	0.0745 (15)	−0.0107 (10)	0.0220 (11)	0.0034 (11)
C10	0.132 (3)	0.0769 (14)	0.098 (2)	0.0380 (17)	−0.026 (2)	−0.0258 (14)
C11	0.0945 (15)	0.0814 (13)	0.121 (2)	−0.0267 (10)	0.0057 (14)	0.0203 (13)
C12	0.0559 (9)	0.0510 (8)	0.0424 (10)	−0.0014 (6)	−0.0027 (7)	0.0088 (8)
C13	0.0541 (9)	0.0598 (9)	0.0496 (11)	−0.0062 (7)	−0.0040 (8)	0.0024 (8)
C14	0.0525 (9)	0.0445 (8)	0.0464 (10)	−0.0003 (6)	−0.0049 (7)	−0.0009 (7)
C15	0.0525 (9)	0.0602 (9)	0.0544 (11)	0.0090 (7)	−0.0011 (8)	0.0079 (8)
C16	0.0638 (11)	0.0705 (10)	0.0619 (12)	0.0078 (8)	0.0060 (9)	0.0169 (9)
C17	0.0697 (11)	0.0565 (9)	0.0527 (11)	0.0068 (8)	−0.0113 (9)	0.0048 (8)
C18	0.0590 (11)	0.1087 (15)	0.0901 (16)	−0.0019 (10)	−0.0229 (11)	0.0293 (13)
C19	0.0559 (11)	0.1068 (14)	0.0806 (15)	−0.0106 (9)	−0.0092 (10)	0.0308 (12)
C20	0.0637 (9)	0.0546 (9)	0.0392 (10)	0.0039 (7)	−0.0034 (8)	0.0036 (7)
C21	0.0785 (11)	0.0597 (9)	0.0562 (12)	−0.0025 (8)	−0.0077 (9)	−0.0014 (8)
C22	0.1206 (16)	0.0589 (11)	0.0655 (14)	0.0083 (10)	−0.0084 (12)	−0.0098 (10)
C23	0.1229 (17)	0.0889 (13)	0.0664 (15)	0.0319 (12)	0.0108 (13)	−0.0087 (11)
C24	0.0745 (11)	0.0834 (12)	0.0579 (13)	0.0147 (9)	0.0083 (10)	0.0063 (10)

### Geometric parameters (Å, °)

**Table:**

Cl1—C17	1.7334 (17)	C20—C24	1.527 (2)
O1—C1	1.361 (2)	C21—C22	1.524 (2)
O1—C20	1.4633 (19)	C22—C23	1.508 (3)
O2—C1	1.202 (2)	C23—C24	1.530 (2)
O3—C12	1.3807 (19)	C5—H5	0.930
O3—C13	1.3681 (18)	C7—H7	0.930
O4—C13	1.195 (2)	C9—H91	0.960
C1—C2	1.475 (2)	C9—H92	0.960
C2—C3	1.483 (2)	C9—H93	0.960
C2—C12	1.317 (2)	C10—H101	0.960
C3—C4	1.393 (2)	C10—H102	0.960
C3—C8	1.396 (2)	C10—H103	0.960
C4—C5	1.389 (2)	C11—H111	0.960
C4—C9	1.507 (2)	C11—H112	0.960
C5—C6	1.386 (2)	C11—H113	0.960
C6—C7	1.369 (3)	C15—H15	0.930
C6—C10	1.524 (3)	C16—H16	0.930
C7—C8	1.383 (2)	C18—H18	0.930
C8—C11	1.504 (2)	C19—H19	0.930
C12—C20	1.4978 (19)	C21—H211	0.970
C13—C14	1.471 (2)	C21—H212	0.970
C14—C15	1.366 (2)	C22—H221	0.970
C14—C19	1.380 (2)	C22—H222	0.970
C15—C16	1.377 (2)	C23—H231	0.970
C16—C17	1.368 (2)	C23—H232	0.970
C17—C18	1.357 (2)	C24—H241	0.970
C18—C19	1.378 (2)	C24—H242	0.970
C20—C21	1.520 (2)		
			
C1—O1—C20	110.04 (11)	C6—C5—H5	119.0
C12—O3—C13	117.84 (11)	C6—C7—H7	118.5
O1—C1—O2	121.16 (14)	C8—C7—H7	118.5
O1—C1—C2	109.66 (14)	C4—C9—H91	109.5
O2—C1—C2	129.18 (15)	C4—C9—H92	109.5
C1—C2—C3	123.33 (15)	C4—C9—H93	109.5
C1—C2—C12	105.54 (13)	H91—C9—H92	109.5
C3—C2—C12	131.13 (14)	H91—C9—H93	109.5
C2—C3—C4	119.47 (13)	H92—C9—H93	109.5
C2—C3—C8	119.99 (14)	C6—C10—H101	109.5
C4—C3—C8	120.52 (14)	C6—C10—H102	109.5
C3—C4—C5	118.58 (15)	C6—C10—H103	109.5
C3—C4—C9	121.32 (14)	H101—C10—H102	109.5
C5—C4—C9	120.11 (16)	H101—C10—H103	109.5
C4—C5—C6	121.98 (19)	H102—C10—H103	109.5
C5—C6—C7	117.69 (18)	C8—C11—H111	109.5
C5—C6—C10	120.8 (2)	C8—C11—H112	109.5
C7—C6—C10	121.5 (2)	C8—C11—H113	109.5
C6—C7—C8	122.96 (18)	H111—C11—H112	109.5
C3—C8—C7	118.25 (17)	H111—C11—H113	109.5
C3—C8—C11	121.71 (15)	H112—C11—H113	109.5
C7—C8—C11	120.04 (16)	C14—C15—H15	119.7
O3—C12—C2	128.10 (12)	C16—C15—H15	119.7
O3—C12—C20	118.17 (13)	C15—C16—H16	120.2
C2—C12—C20	113.69 (13)	C17—C16—H16	120.2
O3—C13—O4	121.26 (15)	C17—C18—H18	120.1
O3—C13—C14	112.40 (13)	C19—C18—H18	120.1
O4—C13—C14	126.30 (15)	C14—C19—H19	119.8
C13—C14—C15	122.92 (13)	C18—C19—H19	119.8
C13—C14—C19	117.99 (14)	C20—C21—H211	111.2
C15—C14—C19	119.05 (15)	C20—C21—H212	111.2
C14—C15—C16	120.62 (14)	C22—C21—H211	111.2
C15—C16—C17	119.63 (15)	C22—C21—H212	111.2
Cl1—C17—C16	120.17 (13)	H211—C21—H212	109.5
Cl1—C17—C18	119.25 (13)	C21—C22—H221	110.9
C16—C17—C18	120.53 (16)	C21—C22—H222	110.9
C17—C18—C19	119.79 (16)	C23—C22—H221	110.9
C14—C19—C18	120.34 (16)	C23—C22—H222	110.9
O1—C20—C12	101.01 (11)	H221—C22—H222	109.5
O1—C20—C21	108.02 (12)	C22—C23—H231	110.3
O1—C20—C24	109.27 (12)	C22—C23—H232	110.3
C12—C20—C21	115.86 (12)	C24—C23—H231	110.3
C12—C20—C24	117.98 (12)	C24—C23—H232	110.3
C21—C20—C24	104.32 (12)	H231—C23—H232	109.5
C20—C21—C22	102.49 (13)	C20—C24—H241	110.3
C21—C22—C23	103.88 (14)	C20—C24—H242	110.3
C22—C23—C24	106.19 (16)	C23—C24—H241	110.3
C20—C24—C23	106.01 (14)	C23—C24—H242	110.3
C4—C5—H5	119.0	H241—C24—H242	109.5
			
C1—O1—C20—C12	1.70 (14)	C5—C6—C7—C8	−1.2 (3)
C1—O1—C20—C21	−120.35 (12)	C10—C6—C7—C8	178.4 (2)
C1—O1—C20—C24	126.75 (12)	C6—C7—C8—C3	0.1 (2)
C20—O1—C1—O2	179.74 (14)	C6—C7—C8—C11	179.8 (2)
C20—O1—C1—C2	−0.57 (16)	O3—C12—C20—O1	175.69 (11)
C12—O3—C13—O4	−5.0 (2)	O3—C12—C20—C21	−67.91 (18)
C12—O3—C13—C14	176.84 (12)	O3—C12—C20—C24	56.74 (18)
C13—O3—C12—C2	−71.27 (19)	C2—C12—C20—O1	−2.47 (16)
C13—O3—C12—C20	110.86 (14)	C2—C12—C20—C21	113.92 (16)
O1—C1—C2—C3	178.86 (12)	C2—C12—C20—C24	−121.43 (16)
O1—C1—C2—C12	−1.01 (16)	O3—C13—C14—C15	−9.4 (2)
O2—C1—C2—C3	−1.5 (2)	O3—C13—C14—C19	168.51 (14)
O2—C1—C2—C12	178.65 (16)	O4—C13—C14—C15	172.61 (16)
C1—C2—C3—C4	−70.0 (2)	O4—C13—C14—C19	−9.5 (2)
C1—C2—C3—C8	108.53 (18)	C13—C14—C15—C16	179.61 (14)
C1—C2—C12—O3	−175.75 (13)	C13—C14—C19—C18	−179.44 (16)
C1—C2—C12—C20	2.20 (17)	C15—C14—C19—C18	−1.5 (2)
C3—C2—C12—O3	4.4 (2)	C19—C14—C15—C16	1.7 (2)
C3—C2—C12—C20	−177.65 (14)	C14—C15—C16—C17	−0.2 (2)
C12—C2—C3—C4	109.8 (2)	C15—C16—C17—Cl1	−178.99 (12)
C12—C2—C3—C8	−71.6 (2)	C15—C16—C17—C18	−1.7 (2)
C2—C3—C4—C5	178.81 (15)	Cl1—C17—C18—C19	179.29 (14)
C2—C3—C4—C9	−1.5 (2)	C16—C17—C18—C19	1.9 (2)
C2—C3—C8—C7	−178.18 (17)	C17—C18—C19—C14	−0.4 (2)
C2—C3—C8—C11	2.1 (2)	O1—C20—C21—C22	−78.01 (15)
C4—C3—C8—C7	0.4 (2)	O1—C20—C24—C23	94.02 (15)
C4—C3—C8—C11	−179.37 (17)	C12—C20—C21—C22	169.61 (14)
C8—C3—C4—C5	0.3 (2)	C12—C20—C24—C23	−151.47 (14)
C8—C3—C4—C9	179.99 (14)	C21—C20—C24—C23	−21.28 (17)
C3—C4—C5—C6	−1.4 (2)	C24—C20—C21—C22	38.17 (16)
C9—C4—C5—C6	178.88 (19)	C20—C21—C22—C23	−40.81 (18)
C4—C5—C6—C7	1.8 (3)	C21—C22—C23—C24	27.7 (2)
C4—C5—C6—C10	−177.7 (2)	C22—C23—C24—C20	−4.02 (19)

### Hydrogen-bond geometry (Å, °)

**Table:**

*D*—H···*A*	*D*—H	H···*A*	*D*···*A*	*D*—H···*A*
C24—H242···O2^i^	0.97	2.57	3.475 (2)	155

Symmetry codes: (i) *x*, −*y*+1/2, *z*−1/2.
